# 1394. Autochthonous Leprosy in Missouri

**DOI:** 10.1093/ofid/ofab466.1586

**Published:** 2021-12-04

**Authors:** Paragkumar Patel, Christian Rojas-Moreno, Taylor Nelson, Araya Zaesim, Jon Dyer

**Affiliations:** University of Missouri- Columbia, Columbia, Missouri

## Abstract

**Background:**

Introduction: Leprosy (Hansen’s disease) is a chronic granulomatous infection of the skin/peripheral nerves caused by Mycobacterium leprae. Of 216 new cases reported in the US in 2019, 70% were in FL, LA, TX, HI, CA, GA and NY. Leprosy is considered a zoonosis in the southern US with the nine-banded armadillo as a reservoir. There have been no reported autochthonous leprosy cases in Missouri.

**Methods:**

Case: 55 y/o previously healthy male noted a new rash on his arm 2 years ago. Over time it spread to his extremities/torso. Skin biopsy showed a granulomatous infiltrate, suspected granuloma annulare, but it progressed despite appropriate therapy. He noted progressive numbness of the affected areas of skin and several regional nerve distributions. In the weeks prior to his initial visit he noted facial swelling, eyelid and ear induration, worsening fatigue, diffuse arthralgia, and some vision changes. His travel history is limited to Canada, Colorado and a brief vacation to the Texas/Mexico border (no notable outdoor exposure during the latter trip; no travel outside the country). He lives in rural Missouri where he is exposed to armadillos. His dogs frequently kill them and often bring them into the yard, rolling around on/in the dead carcasses which he disposes of. He typically wears gloves when handling them and has never consumed them. On exam he had diffuse purplish-red nummular infiltrated anesthetic papules and plaques diffusely distributed over the trunk and extremities. Distinct left ulnar neuropathy was noted. He exhibited leonine facies and infiltration of the bilateral helices. Repeat biopsy showed a granulomatous infiltrate with abundant acid-fast bacilli. DNA sequencing confirmed M. leprae. He was preventatively treated with prednisone and methotrexate to minimize immune reaction, and two weeks later began a regimen of monthly rifampin, minocycline, and moxifloxacin with an anticipated duration of 24 months.

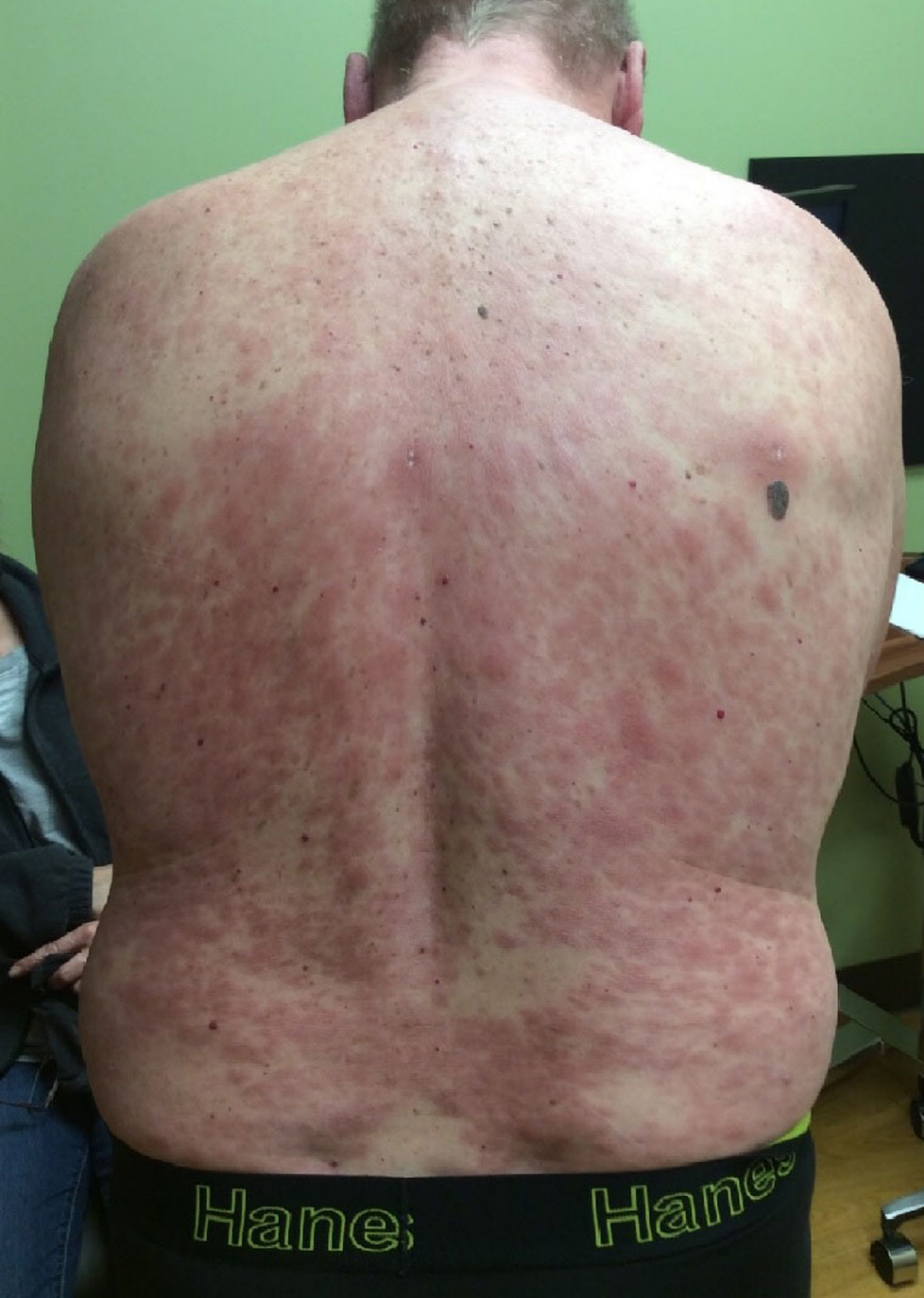

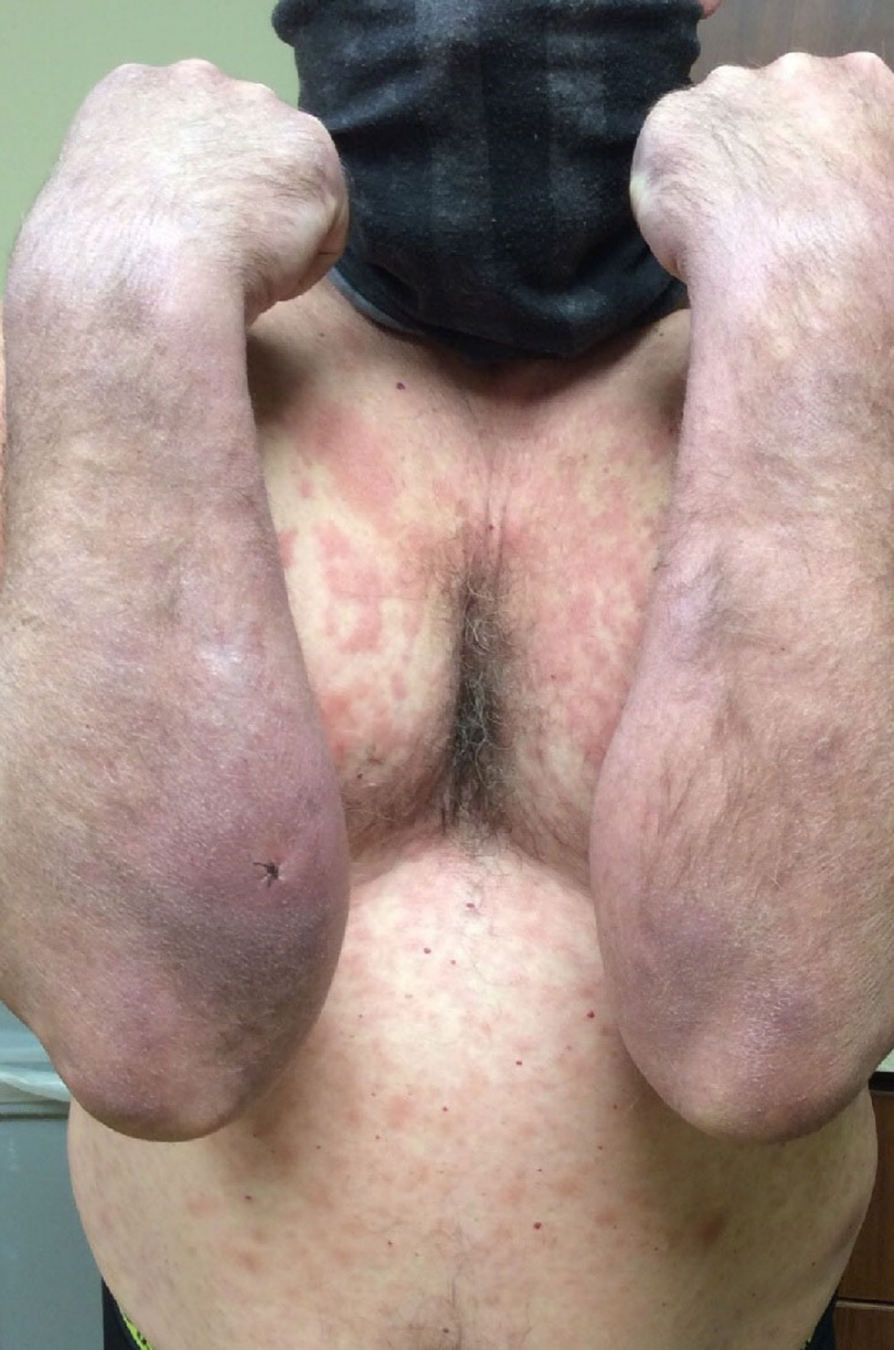

Results

**Diagnosis:**

lepromatous leprosy with pre-treatment immune reaction. This is potentially the first case of autochthonous leprosy in Missouri. Providers should include Hansen’s disease in the differential diagnosis of patients with dermal eruption and cutaneous neurological symptoms to avoid delays in diagnosis/care.

**Disclosures:**

**All Authors**: No reported disclosures

